# Metoclopramide-Induced Akathisia Mimicking Seizure With Transient Lactic Acidosis

**DOI:** 10.7759/cureus.106911

**Published:** 2026-04-12

**Authors:** Adrian Rodriguez Hernandez, Brittany Dellechiaie

**Affiliations:** 1 Neurology, AdventHealth Florida, Orlando, USA

**Keywords:** akathisia, drug-induced movement disorders, extrapyramidal side effects, metoclopramide, seizure mimic

## Abstract

Metoclopramide is a commonly used antiemetic associated with extrapyramidal side effects, including akathisia. Although typically mild, severe presentations may mimic neurologic emergencies. We present an 81-year-old male with chronic obstructive pulmonary disease (COPD) admitted for respiratory syncytial virus (RSV) pneumonia who developed acute severe akathisia after a single dose of metoclopramide. The episode began with acute back pain followed by marked restlessness and nonrhythmic movements with preserved awareness, favoring a toxic-metabolic process over seizure. Laboratory evaluation revealed transient lactic acidosis (6.3 mmol/L), further confounding the diagnosis. Despite treatment with diphenhydramine and lorazepam, symptoms progressed, requiring intensive care unit (ICU) transfer due to worsening agitation and decreased awareness. Symptoms resolved completely within 24 hours. This case highlights the importance of recognizing medication-induced akathisia, which can mimic a seizure and lead to escalation of care, particularly in elderly patients with concurrent infection.

## Introduction

Metoclopramide, a dopamine D2 receptor antagonist, is widely used for nausea and gastroparesis and is associated with extrapyramidal side effects due to central dopaminergic blockade [1,2]. These include akathisia, dystonia, and Parkinsonism. Akathisia is characterized by subjective inner restlessness and observable motor agitation and is frequently underrecognized in acute care settings [3]. While typically associated with repeated dosing, acute akathisia can occur even after a single administration, particularly in elderly patients or those with multiple comorbidities [4].

Severe presentations may mimic seizure, delirium, or other neurologic emergencies, creating diagnostic uncertainty. In medically complex patients, overlapping conditions such as infection or metabolic derangements further complicate recognition [3,5]. We report a case of severe akathisia following a single dose of metoclopramide, complicated by transient lactic acidosis, encephalopathy, and ICU transfer.

## Case presentation

An 81-year-old male with a history of COPD, dyslipidemia, benign prostatic hyperplasia, and sciatica presented with cough, fever, and shortness of breath. He was diagnosed with RSV-positive pneumonia and COPD exacerbation. He also reported a one- to two -month history of new-onset headaches following a left-sided fungal otologic infection requiring foreign body removal.

On admission, neurological examination revealed no focal deficits. Computed tomography (CT) of the head demonstrated no acute intracranial abnormality. The patient was admitted for management of RSV pneumonia and COPD exacerbation and was treated with supportive care, including bronchodilator therapy (albuterol/ipratropium), doxycycline, and systemic corticosteroids (prednisone 40 mg). His home medications, including atorvastatin and tamsulosin, were continued.

On hospital day two, for symptomatic management of a severe headache associated with nausea, the patient received a single dose of 10 mg intravenous metoclopramide, along with intravenous acetaminophen and magnesium. The intravenous route was selected to provide rapid symptom relief in the acute care setting.

Metoclopramide was administered at approximately 6:00 PM. Within minutes, the patient developed acute back discomfort followed by severe restlessness and nonrhythmic, nonstereotyped movements involving the trunk and extremities. He exhibited rocking, swaying, and repetitive crossing and uncrossing of the legs. He remained awake and fully oriented during this initial phase, without gaze deviation, incontinence, or postictal confusion.

Laboratory evaluation obtained approximately one hour after symptom onset revealed an elevated lactic acid level of 6.3 mmol/L. Repeat testing approximately four hours later demonstrated normalization to 1.7 mmol/L as symptoms improved. Hemodynamic parameters remained stable.

The patient was treated with lorazepam (total 2.5 mg) and diphenhydramine (50 mg). Following administration of these medications, he developed confusion, likely reflecting medication-related sedation in the context of infection and physiologic stress. Due to progressive agitation and decreased awareness, he was transferred to the ICU and started on dexmedetomidine for agitation control.

EEG was not available during the acute event. An EEG performed the following morning did not demonstrate epileptiform discharges or focal abnormalities.

Further workup, including repeat imaging and infectious evaluation, revealed no new abnormalities. Blood cultures remained negative, and chest imaging was stable. By the following day, his abnormal movements and mental status had completely returned to baseline without recurrence. The episode was attributed to severe acute akathisia secondary to metoclopramide, with superimposed toxic-metabolic encephalopathy.

**Figure 1:**
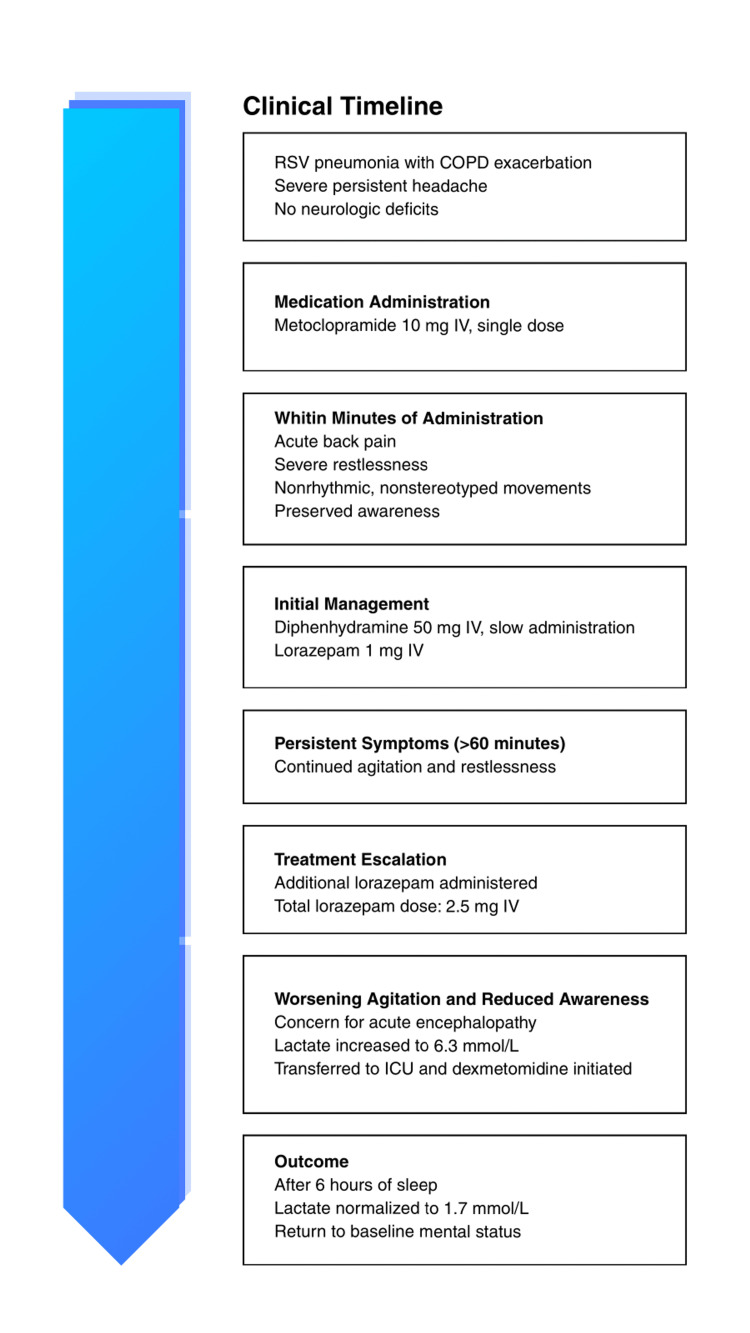
Clinical timeline of metoclopramide induced akathisia mimicking seizure Clinical timeline of symptoms, management, and outcome following metoclopramide administration. RSV - respiratory syncytial virus; COPD - chronic obstructive pulmonary disease; IV - intravenous; ICU - intensive care unit

The clinical course and temporal relationship of events are summarized in Figure 1.

## Discussion

Akathisia is a well-recognized adverse effect of dopamine antagonists such as metoclopramide and is characterized by subjective restlessness and observable motor agitation [3]. Although often mild, severe cases may present dramatically and mimic neurologic emergencies, including seizures or acute encephalopathy [5].

First, akathisia can occur after a single dose of metoclopramide, particularly in elderly patients who may have increased sensitivity to dopamine blockade [4]. Prior studies in emergency department settings have reported akathisia rates of approximately 11% to 25% following rapid intravenous bolus administration of metoclopramide, with significantly lower rates observed when the drug is administered as a slow infusion [6,7]. Increased susceptibility in elderly patients is likely multifactorial, including both enhanced central nervous system sensitivity to dopaminergic blockade and age-related pharmacokinetic changes such as reduced clearance and altered volume of distribution [8].

Second, the clinical presentation closely mimicked seizure activity; however, preserved awareness during the initial phase, nonrhythmic and nonstereotyped movements, and absence of a postictal state argue against an epileptic etiology and favor a hyperkinetic movement disorder such as akathisia. The differential diagnosis also included acute dystonia, delirium, and toxic-metabolic encephalopathy. The observed restlessness with rocking, swaying, and repetitive leg movements is more consistent with akathisia, while the absence of sustained muscle contractions or fixed posturing argues against dystonia. Confusion developed later in the course after administration of lorazepam and diphenhydramine and is most consistent with medication-related sedation in the context of infection and physiologic stress [5,9].

Third, the presence of transient lactic acidosis added diagnostic complexity. While elevated lactate is commonly associated with generalized seizures, it can also result from intense skeletal muscle activity and adrenergic stimulation, both of which are present in severe akathisia. In this case, the findings are most consistent with activity-induced (muscle-derived) lactic acidosis, a form of type B lactic acidosis [10]. Sustained muscle contractions and agitation increase anaerobic metabolism, leading to lactate accumulation. Rapid normalization of lactate further supports a transient, activity-related process rather than ongoing tissue hypoxia or sepsis.

A structured causality assessment using the Naranjo Adverse Drug Reaction Probability Scale yielded a score of 6, consistent with a probable adverse drug reaction [11]. The temporal relationship between metoclopramide administration and symptom onset, absence of alternative causative medications, and complete resolution after discontinuation support metoclopramide as the likely trigger.

Although benzodiazepines such as lorazepam are commonly used in the management of akathisia, their effect may be limited as they do not directly reverse dopaminergic blockade [5]. In this case, the response appeared limited, likely due to the severity of symptoms and subsequent development of medication-related encephalopathy after lorazepam and diphenhydramine, which may have obscured the observable therapeutic effect.

The complete resolution of symptoms within 24 hours is consistent with the pharmacokinetics of metoclopramide, which has an elimination half-life of approximately five to six hours [12]. As drug levels decline, extrapyramidal symptoms typically improve following discontinuation [1].

This case has several limitations. A standardized assessment tool such as the Barnes Akathisia Rating Scale (BARS) was not utilized, limiting objective quantification of symptom severity. Additionally, an EEG was not available during the acute event, which limits the ability to definitively exclude nonconvulsive status epilepticus at symptom onset. Furthermore, the patient received lorazepam and diphenhydramine during the acute episode, which may have influenced the clinical presentation and contributed to subsequent encephalopathy. Finally, given the presence of concurrent infection and systemic illness, a multifactorial contribution to the patient's presentation cannot be entirely excluded.

## Conclusions

Severe akathisia can occur even after a single dose of metoclopramide and may closely mimic neurologic emergencies such as seizures or acute encephalopathy, particularly in elderly patients with concurrent infection. Careful medication review and close attention to the temporal relationship between drug administration and symptom onset are critical for accurate diagnosis. Recognition of distinguishing features, including preserved awareness and nonrhythmic movements, can help differentiate akathisia from seizure and prevent unnecessary escalation of care. Early identification and prompt discontinuation of the offending agent, along with appropriate symptomatic management, are essential to optimize outcomes.
